# Serum Matrix Metalloproteinase-3 as a Noninvasive Biomarker of Histological Synovitis for Diagnosis of Rheumatoid Arthritis

**DOI:** 10.1155/2014/179284

**Published:** 2014-07-23

**Authors:** Jian-Da Ma, Jing-Jing Zhou, Dong-Hui Zheng, Le-Feng Chen, Ying-Qian Mo, Xiu-ning Wei, Li-Juan Yang, Lie Dai

**Affiliations:** Department of Rheumatology, Sun Yat-Sen Memorial Hospital, Sun Yat-Sen University, No. 107 Yan Jiang West Road, Guangzhou 510120, China

## Abstract

*Objective*. To explore the correlation between matrix metalloproteinase- (MMP-) 3 and histological synovitis in rheumatoid arthritis (RA). *Methods*. Serum MMP-3 of 62 patients with active RA was detected by ELISA. Serial synovial tissue sections from all RA patients, 13 osteoarthritis, and 10 orthopedic arthropathies patients were stained with hematoxylin and eosin and immunohistochemically for MMP-3, CD3, CD20, CD38, CD68, and CD15. *Results*. The percentage of lining MMP3+ cells was significantly higher in RA patients especially with high grade synovitis and it was significantly correlated with Krenn's synovitis score (*r* = 0.574, *P* < 0.001) and sublining inflammatory cells. Multivariate stepwise linear regression analysis revealed that the association of the percentage of lining MMP3+ cells with activation of synovial stroma, sublining CD68+ macrophages, and CD15+ neutrophils was stronger than other histological indicators. The percentage of lining MMP3+ cells was significantly correlated with serum MMP-3 in RA (*r* = 0.656, *P* < 0.001). Serum MMP-3 was higher in RA patients with high grade synovitis than that of low grade synovitis and significantly correlated with synovitis score and activation of synovial stroma subscore (all *P* < 0.05). *Conclusion*. Serum MMP-3 may be an alternative noninvasive biomarker of histological synovitis and RA diagnosis.

## 1. Introduction

Rheumatoid arthritis (RA) is an autoimmune disease characterized by chronic synovitis which leads to cartilage degradation, subchondral bone erosions, and eventual disability [1]. Traditional and novel assessment tools for synovitis include physical examination, imaging techniques, histological synovitis analysis, and serological biomarkers. As histopathological features are the golden standard for diagnosis of synovitis, histological synovitis analysis is a powerful tool for clarifying the pathological change in synovium and immunochemical analysis of synovitis is helpful for the study of pathogenesis and prognostic evaluation [2, 3].

Histopathological features of RA synovitis include hyperplasia of lining layer, inflammatory infiltration (mostly lymphocytes and plasma cells), and increased density of resident cells (fibrocytes, fibroblasts, and endothelial cells). Several grading systems have been developed for scoring the histopathological features of synovitis in RA, which are useful in providing information combined with morphological and molecular data and giving basic and standardized diagnostic information concerning the inflammatory character of the diseases and the degree of inflammation [4–7]. However, synovial tissue should be obtained by invasive techniques such as arthroscopy or blind needle biopsy, leading to limited histological measures in routine clinical practice. Alternative noninvasive measures such as serological biomarkers are required for synovitis assessment in daily clinical use.

Matrix metalloproteinases (MMPs) are a large group of zinc-dependent proteases with the ability of degrading components of extracellular matrix such as collagen, elastin, gelatin, and casein. MMP-3 is one of the MMP family members produced in joints. It can aggravate inflammation via activating various pro-MMPs such as pro-MMP-1, pro-MMP-7, pro-MMP-8, pro-MMP-9, and pro-MMP-13 and cleave extracellular components including collagen types III, IX, and X and telopeptides of collagen types I, II, and XI [8]. As a secreted MMP, serum MMP-3 level is reported to be increased and positively correlated with inflammatory mediators and disease activity in RA [9]. However, little was known about the relationship between MMP-3 and histological synovitis in RA. Here, we explored the correlation of serum MMP-3 or synovial MMP-3 expression with histological synovitis and the significance for the diagnosis of RA.

## 2. Materials and Methods

### 2.1. Patients

Sixty-two Chinese patients with RA who fulfilled the 1987 revised criteria of the American College of Rheumatology (ACR) [10] or 2010 ACR/EULAR classification criteria for RA [11] were included from the Department of Rheumatology of Sun Yat-sen Memorial Hospital. All patients had active disease, defined as simplified disease activity index (SDAI) >3.3 [12]. Thirteen patients with osteoarthritis (OA) and ten patients with noninflammatory orthopedic arthropathies (OrthA; consisting of discoid meniscus (*n* = 1), meniscus injury (*n* = 5), meniscocapsular tear (*n* = 1), traumatic arthritis (*n* = 1), and anterior cruciate ligament injuries (*n* = 2)) were included from the Department of Orthopedics as “less inflamed” disease controls [13]. Thirty-four age and gender matched healthy individuals were included as controls. This study was conducted in compliance with the Helsinki Declaration. The Medical Ethics Committee of Sun Yat-sen Memorial Hospital approved the protocol. All patients gave written informed consent.

### 2.2. Clinical Assessments

Clinical data of all patients with RA were collected at baseline, including the 28-joint tender and swollen joint count (28TJC and 28SJC), patient global assessment of disease activity (PtGA), provider global assessment of disease activity (PrGA), pain visual analogue scale (Pain VAS), Chinese language version of Stanford Health Assessment Questionnaire (HAQ) [14], erythrocyte sedimentation rate (ESR), C-reactive protein (CRP); rheumatoid factor (RF), and anticyclic citrullinated peptide antibody (anti-CCP). Disease activity was assessed with SDAI, clinical disease activity index (CDAI), and disease activity score in 28 joints (DAS28) with four variables including CRP (DAS28 (4)-CRP) [12].

### 2.3. Serum MMP-3 Detection by ELISA

Serum samples were collected from all RA patients and 34 healthy controls after overnight fasting and stored at −80°C until analysis. Serum level of soluble MMP-3 was measured with a human MMP-3 detection kit (AESKU Diagnostics, Germany) according to the manufacturer's instructions. This detects total MMP-3 (pro- and active MMP-3) in human serum. Measurements were done in duplicate. Serum samples were placed in designated microwells. In addition, calibrators, negative, and positive controls were added to the designated microwells to construct a standard curve. The plates were then incubated for 30 min at 26°C and washed with wash buffer 3 times. Then 100 *μ*L TMB substrate was added to each well and incubated for 30 min at 26°C, protected from intense light. Then 100 *μ*L of stop solution was added to each well, using the same order as added the substrate, and incubated for 5 minutes minimum. The absorbance of each well was read at 450 nm (optionally 450/620 nm) within 30 min. The normal ranges of serum MMP-3 concentrations were 18–60 ng/mL (female) or 24–120 ng/mL (male). The assays were performed blindly, without knowledge of the patient's disease status or activity.

### 2.4. Synovial Tissue

Closed Parker-Pearson needle biopsy was performed on knee joints of all patients with RA [15]. At least 6 pieces of synovial tissue were obtained per patient to minimize sampling error [16]. The OA and OrthA specimens were obtained from knees by closed needle biopsy, arthroplasty, or arthroscopy. All samples were fixed in 10% neutral formalin and embedded in paraffin. Sections (5 *μ*m) were cut serially and mounted on adhesive glass slides. Sealed slides were stored at −20°C until staining.

### 2.5. Immunohistochemistry

Serial sections of synovial tissues were stained with hematoxylin and eosin (H and E) and a 3-step immunoperoxidase method. Sections were deparaffinized with xylol, ethanol, and demineralized water. Antigens were then retrieved by boiling in 1 mM EDTA (pH 8.0) for 10 to 15 min. After the sections had been washed in demineralized water and phosphate buffered saline (PBS), the primary antibody was added and incubated overnight at 4°C. After washing with PBS, the sections were incubated with EnVision Mouse or Rabbit conjugate (Dako Corporation, Carpinteria, CA, USA) for 30 min at 37°C. The color reaction was completed with the DAB-positive substrate. Sections were counterstained with hematoxylin. Nonspecific isotype IgG was used as a negative control. Absence of staining due to technical failure was excluded by including appropriate positive control tissues (breast carcinoma) in each staining run.

Serial sections were stained with rabbit anti-human MMP-3 (clone EP1186Y, Novus Biological, Littleton, CO, USA) and the following commercial mouse anti-human antibody preparations (Invitrogen, Carlsbad, CA, USA): anti-CD20 (clone L26, B cells), anti-CD38 (clone SPC32, plasma cells), anti-CD3 (clone PS1, T cells), anti-CD68 (clone KP1, macrophage-like synoviocytes and macrophages), and anti-CD15 (clone My1, neutrophils), according to standard staining protocols. All were monoclonal antibodies. Parallel sections were incubated with irrelevant, isotype, and concentration-matched monoclonal antibodies as negative control.

### 2.6. Synovitis Assessments

At least three qualified tissue pieces containing both synovial lining and sublining layers for each specimen were included in the analyses. Histologic changes of RA synovitis in the H and E-stained sections were graded with Krenn's synovitis score [7, 17], which contains three subscores of hyperplasia of lining layer, inflammatory infiltration, and activation of synovial stroma, each was scored from 0 to 3 and the sum provided the synovitis score from 0 to 9. All RA patients were then divided into high grade (>4) or low grade (≤4) synovitis groups according to the synovitis score. Similarly, they were divided into corresponding high grade (>2) or low grade (≤2) groups according to each synovitis subscore. Synovitis assessments were performed by 2 blinded observers (MYQ and MJD) using a Leica DM2500 microscope (Leica Corp. Heidelberg, Germany). Differences between the observers were resolved by mutual agreement.

The percentage of lining MMP-3 and CD68 positive-staining cells was determined by manual counting in the lining layer observing 5 different fields at magnification of ×400. In sublining area, the densities of CD20, CD38, CD3, CD15, or CD68 positive-staining cells were determined by manual counting combined with cellular morphology in consecutive sections stained with H and E. A selection of 9 hpf (400x) of the sublining area was examined for each specimen by two independent observers (MJD and CLF). As one hpf revealed a synovial area of 0.11740 mm^2^, the densities of CD38, CD3, CD20, CD15, or CD68 positive-staining cells were counted as cells per mm^2^ [18].

### 2.7. Statistical Analysis

Statistical analyses were performed with SPSS 13.0 statistical software (SPSS Inc., Chicago, IL, USA). Data of categorical variables were presented as frequencies and percentages. Data of continuous variables were presented as median and interquartile range (IQR). Nonparametric tests (Mann-Whitney ranksum test between two groups or Kruskal-Wallis one-way analysis of variance on ranks among three groups for continuous variables) were used to compare the differences of serum MMP-3, synovial MMP-3, or clinical indicators in different groups, as data of the mentioned variables were not distributed normally. Correlations between serum or synovial MMP-3 expression and clinical or histological indicators were analyzed by Spearman's rank order correlation test. The abilities of serum and synovial MMP-3 to distinguish high grade from low grade synovitis were assessed with receiver operating characteristic curve (ROC) analysis. Multiple linear regression models were developed by stepwise variable selection method to describe the linear association between synovial MMP-3 and histological indicators, including components of synovitis score and densities of sublining inflammatory cells. All significance tests were two-tailed and were conducted at the 5% significance level.

## 3. Results

### 3.1. Characteristics of the Study Patients

Baseline demographic and clinical features of all patients with RA were shown in Table 1. Age and sex did not differ among the patients with RA, OA, and OrthA. Among the patients with RA, 37% (23/62) had never taken corticosteroids or disease modifying antirheumatic drugs (DMARDs). A majority of these 23 patients had taken only Chinese herbal medicine and/or painkillers to relieve arthralgia. At recruitment, 16% (10/62) had taken corticosteroids alone. The remaining 47% (29/62) received treatment of one or more DMARDs, including methotrexate, leflunomide, sulfasalazine, hydroxychloroquine, and etanercept.

### 3.2. Synovial MMP-3 Expression and Its Correlation with Histological Synovitis

**Figure 1:**
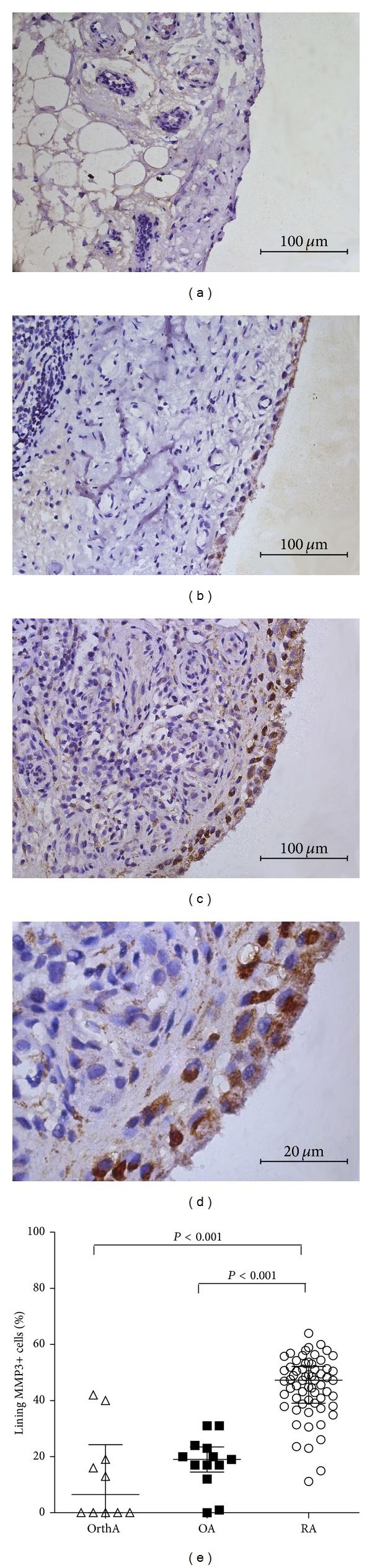
Representative immunohistochemical findings of synovial MMP-3 expression. (a) Mild synovial MMP-3 expression in lining cells in a discoid meniscus patient. (b) Moderate synovial MMP-3 expression in lining cells in an OA patient. (c) and (d) Intensive synovial MMP-3 expression in lining cells in a RA patient. (a, b, c) original magnification ×400; (d) original magnification ×1000. (e) Percentage of lining MMP3+ cells in OrthA, OA, and RA patients.

In synovium, MMP-3 is expressed predominantly in the endochylema of lining cells (both macrophage-like synoviocytes and fibroblast-like synoviocytes), while it is absent in the sublining area. As shown in Figure 1, the percentage of MMP3+ lining cells in RA patients (median 47%, IQR 39~52%) was significantly higher than that in OA (median 19%, IQR 15~24%, *P* < 0.001) or in OrthA patients (median 7%, IQR 0~24%, *P* < 0.001).

**Figure 2:**
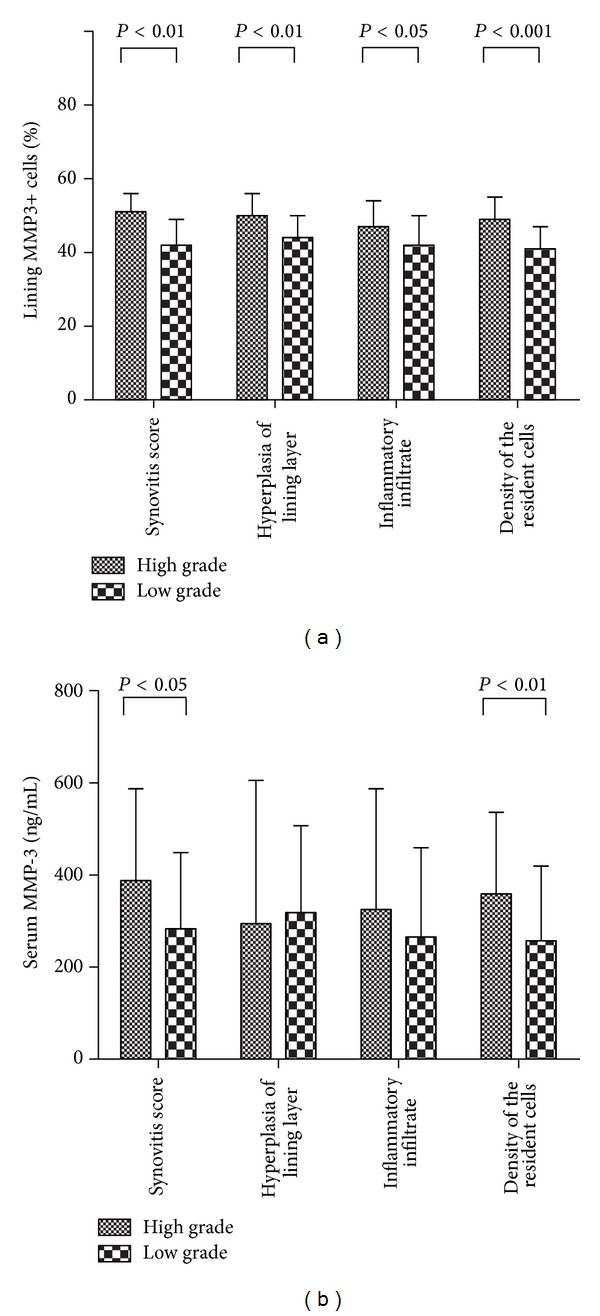
Synovial (a) and serum (b) MMP-3 expression between high and low grade groups of synovitis score or subscore.

**Figure 3:**
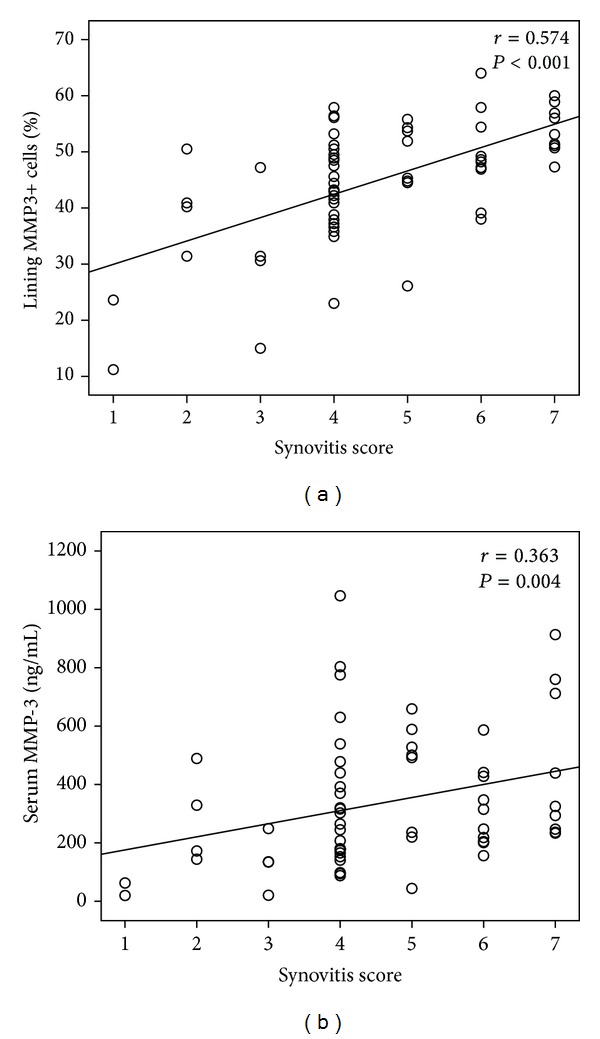
Correlation between synovial (a) and serum (b) MMP-3 with histological synovitis score.

**Figure 4:**
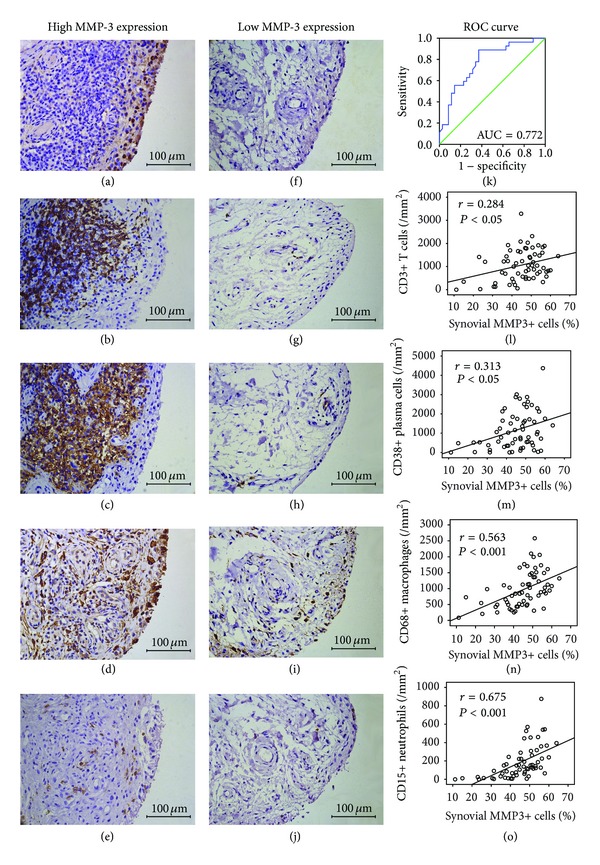
(a~j) Synovial MMP-3 expression and inflammatory cells in representative synovium from 2 different patients with RA. High and low MMP-3 expression in the endochylema of lining cells in RA synovium (a and f). Case one showed aggregated CD3+ T cells (b) and CD38+ plasma cells (c), together with diffuse infiltration of CD68+ macrophages (d) and CD15+ neutrophils (e). Case two showed a small number of CD3+ T cells (g), CD38+ plasma cells (h), CD68+ macrophages (i), and CD15+ neutrophils (j). In panels (a) to (j), immunohistochemical stains with DAB as chromogen (brown); original magnification ×400. (k) ROC curve showed synovial MMP-3 with the ability to distinguish high grade from low grade synovitis. (l~o) Spearman's rank correlation analysis between synovial MMP-3 and CD3+ T cells (l), CD38+ plasma cells (m), CD68+ macrophages (n), and CD15+ neutrophils (o).

The percentage of lining MMP3+ cells was significantly higher in RA patients with high grade synovitis than that in RA patients with low grade synovitis (median 51%, IQR 47%~56% versus median 42%, IQR 36%~49%, *P* < 0.001), and synovial MMP-3 expression was also higher in high grade group of hyperplasia of lining layer, inflammatory infiltration, and activation of synovial stroma (Figure 2(a)). Spearman's rank order correlation test showed significant correlations between the percentage of MMP3+ lining cells and synovitis score (*r* = 0.574), hyperplasia of lining layer subscore (*r* = 0.434), inflammatory infiltration subscore (*r* = 0.287), and activation of synovial stroma subscore (*r* = 0.546), all *P* < 0.05 (Figure 3(a)). ROC curve analysis showed that the tradeoff value of the percentage of lining MMP3+ cells for distinguishing high grade synovitis in RA was 44% with sensitivity 89% and specificity 63% (Table 2 and Figure 4(k)).

According to the tradeoff value of the percentage of lining MMP3+ cells for distinguishing high grade and low grade synovitis, RA patients were divided into high synovial MMP-3 expression (>44%) and low synovial MMP-3 expression groups (≤44%). Densities of CD3+ T cells, CD20+ B cells, CD38+ plasma cells, and CD68+ macrophages in sublining area of synovium in patients with high synovial MMP-3 expression were significantly higher than those in patients with low synovial MMP-3 expression (all *P* < 0.05, Figures 4(a)~4(j)). Further investigation with immunohistochemistry showed that the percentage of MMP3+ lining cells was positively correlated with the density of CD3+ T cells (*r* = 0.284), CD38+ plasma cells (*r* = 0.313), CD68+ macrophages (*r* = 0.563), and CD15+ neutrophils (*r* = 0.675) in sublining area (Figures 4(l)~4(o)).

Multiple linear regression models for synovial MMP-3 were developed using the percentage of lining MMP3+ cells as dependent variable, while components of synovitis score and densities of sublining inflammatory cells correlated with synovial MMP-3 as independent variables, including hyperplasia of lining layer, inflammatory infiltration, activation of synovial stroma, CD3+ T cells, CD38+ plasma cells, CD68+ macrophages, and CD15+ neutrophils. The final model, shown in Table 3, accounted for 54% of total variation in synovial MMP-3. Subscore of activation of synovial stroma, densities of sublining CD68+ macrophages, and CD15+ neutrophils were incorporated in the final model (all *P* < 0.01).

### 3.3. Correlation of Synovial MMP-3 with Serum MMP-3

The level of serum MMP-3 was elevated in RA patients compared with healthy controls (median 257 ng/mL, IQR 174~481 ng/mL versus median 32 ng/mL, IQR 18~53 ng/mL, *P* < 0.001). There was no significant difference between female and male RA patients (median 294 ng/mL, IQR 174~478 ng/mL versus median 249, IQR 165~489 ng/mL, *P* = 0.876). Spearman's rank order correlation test showed significant correlations between the percentage of lining MMP3+ cells and serum MMP-3 (*r* = 0.656, *P* < 0.001), CRP (*r* = 0.378, *P* = 0.002), and ESR (*r* = 0.367, *P* = 0.003). There was no significant correlation between synovial MMP-3 and 28TJC, 28SJC, PtGA, PrGA, HAQ, RF, anti-CCP, DAS28, SDAI, or CDAI (all *P* > 0.05).

### 3.4. Correlation of Serum MMP-3 with Synovitis

Serum MMP-3 in RA patients with high grade synovitis was significantly higher than that in patients with low grade synovitis score (median 325 ng/mL, IQR 233~528 ng/mL versus median 207 ng/mL, IQR 141~392 ng/mL, *P* = 0.024), and serum MMP-3 was also higher in RA patients with high grade group of the resident cells density (Figure 2(b)). Spearman's rank order correlation test showed significant correlations between the serum MMP-3 and synovitis score (*r* = 0.363) and activation of synovial stroma subscore (*r* = 0.351), both *P* < 0.05 (Figure 3(b)). ROC curve analysis showed that the tradeoff value of serum MMP-3 for distinguishing high grade synovitis in RA was 190 ng/mL with sensitivity 93% and specificity 49% (Table 2). There was no significant difference in 28TJC, 28SJC, PtGA, PrGA, HAQ, ESR, CRP, RF, or anti-CCP between high grade and low grade synovitis and no significant correlation between 28TJC, 28SJC, PtGA, PrGA, HAQ, ESR, CRP, RF, anti-CCP, and synovitis score (all *P* > 0.05).

## 4. Discussion

This study provided relatively large scale of synovial tissues for histological analysis of MMP-3 from RA patients. Our results showed that synovial MMP-3 was elevated in RA synovium and positively correlated with synovitis assessed by comprehensive histological analysis including Krenn's synovitis score and inflammatory cells by immunohistochemistry. As a result of correlation test and multiple linear regression analysis, synovial MMP-3 showed stronger association with activation of synovial stroma and sublining infiltration of macrophages and neutrophils than other histological indicators. Serum MMP-3 was significantly correlated with synovial MMP-3 and Krenn's synovitis score and had the ability to distinguish high grade from low grade synovitis in RA.

### 4.1. Serological Biomarkers of Synovitis

RA results in a chronic and systemic inflammatory disorder that may affect many tissues and organs especially joints. Synovial tissues in RA can produce cytokines which aggravate inflammatory response, and uncontrolled synovitis leads to joint damage. Previous studies did not support traditional inflammatory biomarkers and cytokines as biomarkers of synovitis. CRP is a widely used inflammatory biomarker produced by hepatocytes in response to proinflammatory cytokines such as TNF and interleukins during inflammation, but it has poor value in the assessment of cumulative synovitis [19]. There was no agreement on the correlation with persistent synovitis and cytokines such as IL-1, IL-6, and TNF-*α* [20, 21]. Thus, novel serological biomarkers reflecting more specific aspects of histological synovitis and differently regulated in different patients are needed. In this study, we performed histological synovitis analysis to investigate the potential of MMP-3 as a serological biomarker of synovitis, which is an enzyme synthesized in the synovium, secreted into synovial fluids in joint cavities, and then gains access into peripheral circulation [22].

### 4.2. MMP-3 Correlated with Inflammatory Infiltration and Cellular Proliferation

Inflammatory infiltration is one of the main histopathological features in RA synovium. Kobayashi et al. has reported positive correlation between synovial MMP-3 and semiquantitative score of synovial inflammatory cell infiltration [23]. In this study, we further analyzed by immunohistochemistry and found that synovial MMP-3 was positively correlated with inflammatory infiltration subscore and the density of T cells, plasma cells, macrophages, or neutrophils in sublining area, among which macrophages and neutrophils were incorporated in the multiple linear regression model for synovial MMP-3. T cells are abundant in RA synovium and mediate activation of macrophages and fibroblasts [24]. Rat fibroblast-like synoviocytes cocultured with pristane-primed T cells showed upregulation of MMP-3 [25]. Plasma cells are widely distributed in the synovium in RA and secret cytokines, such as TNF-*α*, which can activate MMP-3 production [24]. Macrophages infiltrated in sublining area of RA synovium have been reported to correlate with tissue inflammation and systemic disease activity [26]. In parallel experiments with cultured rheumatoid synovial cells, MMP-3 was detected only in cells treated with macrophage conditioned medium [27]. Neutrophils migrate to the synovial fluid or infiltrate in RA synovium, releasing powerful proteases at the early stage of RA [24]. Increased expression of CD147 on neutrophils in RA may be responsible for elevated MMPs secretion in fibroblast-like synoviocytes [28].

Cellular proliferation is another histopathological feature of synovitis in RA, which includes hyperplasia of lining layer and activation of synovial stroma. Hyperplasia of lining layer always combines with promoting fibroblast-like synoviocytes adhesion, invasion, and synthesis of MMPs [29]. Activation of synovial stroma mostly behaves as increased densities of fibroblasts and endothelial cells in sublining area of synovium, which are activated by cytokines during the joint inflammation. Less reports focusing on cellular proliferation of histological synovitis have been published. Study of the diagnostic accuracy of Krenn's synovitis score showed that the diagnostic power of high grade and low grade synovitis of this score stems more from measuring proliferative than inflammatory features [30]. Using Krenn's synovitis score, we found that synovial MMP-3 was significantly correlated with hyperplasia of lining layer subscore and activation of synovial stroma subscore, and activation of synovial stroma was incorporated in the multiple linear regression model for synovial MMP-3.

### 4.3. Serum MMP-3 as a Serological Biomarker of Synovitis

Serum MMP-3 was elevated in RA patients and was reported as a predictor of joint destruction in clinical studies [9, 31]. Our previous study also showed that serum MMP-3 was positively correlated with 28TJC, 28SJC, CRP, and ESR which suggested the potential of synovitis assessment [32]. In this study, we found significant correlations between synovial MMP-3 and serum MMP-3, CRP, or ESR and significant correlation between serum MMP-3 and synovitis score with high sensitivity of serum MMP-3 in diagnosing high grade or low grade synovitis by ROC curve. Previous studies of clinical and imaging assessments of synovitis emphasize the importance of repeated assessments of synovitis due to the weak prognostic value with single measure [33–35]. Compared to the device-depended imaging assessment and invasive synovial biopsy, serum MMP-3 is more suitable for repeated assessments of synovitis and it may be an alternative noninvasive biomarker of synovitis for clinical practice.

As mentioned in method, serum or synovial total MMP-3 including pro- and active MMP-3 was detected in this paper. However, only active MMP-3 has the ability to cleave extracellular matrix proteins and to aggravate inflammation. Recently Sun et al. successfully developed an assay measuring active MMP-3 detection in human serum and found that serum active MMP-3 only correlated with inflammatory markers CRP and ESR, but not to other measures of disease burden in RA (DAS, HAQ) which implies that active MMP-3 reflects other aspects of disease than total MMP-3 [36]. Our previous data showed that serum total MMP-3 was correlated with CRP, ESR, and DAS. Further studies on the relationship between serum and synovial active MMP-3 and histological synovitis in RA are needed.

## 5. Conclusion

Our results indicated that MMP-3 was involved in pathogenesis of synovitis especially the activation of synovial stroma and sublining infiltration of macrophages and neutrophils, and serum MMP-3 may be an alternative noninvasive biomarker of histological synovitis which is helpful for diagnosis of RA. Further studies are required to investigate whether serum active MMP-3 is more sensitive and reliable serological biomarker of synovitis than total MMP-3.

## Figures and Tables

**Table 1:** Baseline demographic and clinical features of RA patients.

Characteristic	
Demographic	
Age, yrs, median (IQR)	56 (48~62)
Female, *n* (%)	51 (82)
Disease status	
Disease duration, mo, median (IQR)	30 (12 to 96)
ESR (mm/h), median (IQR)	72 (47~107)
CRP (mg/dL), median (IQR)	3.9 (1.0~5.6)
Rheumatoid factor-positive, *n* (%)	54 (87)
Anti-CCP-positive, *n* (%)	50 (81)
SDAI, median (IQR)	33 (24~44)
CDAI, median (IQR)	29 (20~40)
DAS28, median (IQR)	5.5 (4.6~6.3)
Synovitis score, median (IQR)	4 (4~6)
High grade synovitis, *n* (%)	27 (44)
Previous medications, *n* (%)	
Corticosteroids	26 (42)
Methotrexate	20 (32)
Leflunomide	6 (10)
Sulfasalazine	5 (8)
Hydroxychloroquine	7 (11)
Etanercept	4 (6)

**Table 2:** Areas under the curve (AUC) of synovial and serum MMP-3 as biomarkers for distinguishing high grade synovitis.

High synovitis score versus low synovitis score	AUC	*P*	95% CI	Tradeoff value	Youden's index	Sensitivity %	Specificity %
Synovial MMP-3	0.772	<0.001	0.655~0.890	44%	0.517	88.9	62.9
Serum MMP-3	0.668	0.024	0.532~0.803	190.4 ng/mL	0.412	92.6	48.6

**Table 3:** Multiple linear regression analysis of variables associated with synovial MMP-3.

Variable	Unstandardized coefficients		Standardized coefficients	*t*	*P*	95% CI
	*B*	Std. error				
Components of synovitis score						
Hyperplasia of lining layer	—	—	—	1.767	0.083	—
Inflammatory infiltration	—	—	—	0.361	0.719	—
Activation of synovial stroma	6.226	1.568	0.374	3.970	<0.001	3.086~9.366
Sublining inflammatory cells						
CD3+ T cells	—	—	—	0.948	0.347	—
CD38+ plasma cells	—	—	—	0.528	0.599	—
CD68+ macrophages	0.006	0.002	0.284	3.016	0.004	0.002~0.009
CD15+ neutrophils	0.022	0.006	0.368	3.897	<0.001	0.011~0.033
Constant	26.071	2.645	—	9.857	<0.001	20.775~31.368
